# EcRBPome: a comprehensive database of all known *E. coli* RNA-binding proteins

**DOI:** 10.1186/s12864-019-5755-5

**Published:** 2019-05-22

**Authors:** Pritha Ghosh, Adwait Joshi, Niang Guita, Bernard Offmann, R. Sowdhamini

**Affiliations:** 1 National Centre for Biological Sciences, Tata Institute of Fundamental Research, Bellary Road, Bangalore, Karnataka 560 065 India; 2 Faculty of Science and Technology, University of Nantes, Rue de la Houssinière, BP 92208, 44322 Nantes Cedex 3, France; 3 Present address: International Institute of Molecular and Cell Biology in Warsaw, Księcia Trojdena 4, 02-109 Warsaw, Poland

**Keywords:** RNA-binding proteins, *Escherichia coli*, Database, Genome-wide survey, Proteomes, Cross-genome comparison, Pathotypes

## Abstract

The repertoire of RNA-binding proteins (RBPs) in bacteria play a crucial role in their survival, and interactions with the host machinery, but there is little information, record or characterisation in bacterial genomes. As a first step towards this, we have chosen the bacterial model system *Escherichia coli*, and organised all RBPs in this organism into a comprehensive database named EcRBPome. It contains RBPs recorded from 614 complete *E. coli* proteomes available in the RefSeq database (as of October 2018). The database provides various features related to the *E. coli* RBPs, like their domain architectures, PDB structures, GO and EC annotations etc. It provides the assembly, bioproject and biosample details of each strain, as well as cross-strain comparison of occurrences of various RNA-binding domains (RBDs). The percentage of RBPs, the abundance of the various RBDs harboured by each strain have been graphically represented in this database and available alongside other files for user download. To the best of our knowledge, this is the first database of its kind and we hope that it will be of great use to the biological community.

**Electronic supplementary material:**

The online version of this article (10.1186/s12864-019-5755-5) contains supplementary material, which is available to authorized users.

## Background

RNA-binding proteins (RBPs) are important regulators of cellular function, being involved in processes at the transcriptional, post-transcriptional, translational, as well as post-translational levels. They mediate transport, stabilisation, metabolism and degradation of transcripts within the cell [1]. Hence, a proper understanding of the ‘RBPome’ of an organism is essential.

The complete RBP repertoire of a few model organisms have now been identified by various research groups, including ours [2–5], but the data is not conveniently available to the users due to the lack of proper organisation. The most widely used of the RBP repositories, RBPDB [6], reports experimentally observed RNA-binding sites that have been manually curated from literature, but was last updated in 2012. This database houses information from *H. sapiens*, *M. musculus*, *D. melanogaster* and *C. elegans*, but not from *E. coli*. The ATtRACT database [7], reported in 2016, lists information on 370 RBPs and 1583 consensus RNA-binding motifs, and compiles experimentally validated data from multiple resources, including RBPDB. The latest version (v 3.0) of the sRNATarBase [7, 8] contains more than 750 small RNA (sRNA)-target entries collected from literature and other prediction algorithms.

Here, we report EcRBPome (http://caps.ncbs.res.in/ecrbpome), a comprehensive database of *E. coli* RBPs. The database documents RBPs identified in all complete *E. coli* proteomes (available in the RefSeq database, as of October 2018) by computational sequence search algorithms and methods as described earlier [7–9]. The data presented in EcRBPome has been cross-referenced to other popular protein annotation resources, and also made available for user download as parsable and graphical representation files. We hope that this database will be of immense importance to the microbial, and in general to the biological community and can be the start point for understanding RBP-mediated regulation in various other lesser studied species.

## Construction and content

### Datasets

The overall protocol for data acquisition is described in our previous study [9], in which genome-wide survey (GWS) of RBPs was described, but now for 614 complete *E. coli* proteomes, retrieved from the RefSeq database (October 2018) (please see Additional file 1 for further details on the search method). The start-points for such search methods, were known sequence and structure signatures of RBPs, organised as structure-centric and sequence-centric family Hidden Markov Models (HMMs) [5]. A total of 11,662 putative RBPs could be identified from 614 *E. coli* proteomes studied (Table 1). The RefSeq accession numbers, FASTA sequences, domain compositions and cross-references to other databases of these RBPs have been made available for the users in EcRBPome (‘Browse all RBPs in EcRBPome’ under the Browse menu).

**Table 1:** Table of statistics. The various attributes recorded in EcRBPome

Attribute	Numbers
Number of *Escherichia coli* strains	614
Number of RNA-binding proteins	11,662
Types of RNA-binding domains	325
Average percentage of RBPs in *E. coli* proteomes	6.05

### Implementation

The retrieval of data and manipulation logic at the back-end of EcRBPome has been implemented using CGI-Perl and the interface of the database built on HTML5, CSS, JavaScript, Ajax and JQuery. The basic tables in EcRBPome have been organised as comma-separated text files, and converted to JSon format, for performance improvement through utilities. The display of tables has been implemented using Bootstrap DataTables. The downloadable graphical plots have been generated using R and the interactive bar plots using the CanvasJS library of JavaScript and HTML5.

### Features

#### Browse menu

The users can browse through the list of all the *E. coli* strains present in this database (with links to the assembly, biosample and bioproject details for each strain), all RBPs (with links to the RefSeq page and their downloadable FASTA sequences) and their domain architectures (DAs) [10]. The pathogenic and the non-pathogenic strains have been represented in red and green fonts, respectively. The pathotype and sequence type (ST) information, wherever available, has been provided for these strains [11].

The distribution of various RBDs and DAs (domain pairs) in pathogen-specific and nonpathogen-specific proteins have also been represented in various tables (please see Additional file 1 for more details on the identification of pathogen-specific and nonpathogen-specific proteins). The RBDs, pathogen-specific RBDs and domain pairs, and nonpathogen-specific RBDs and domain pairs have been highlighted in bold, red and green fonts, respectively.

**Fig. 1:**
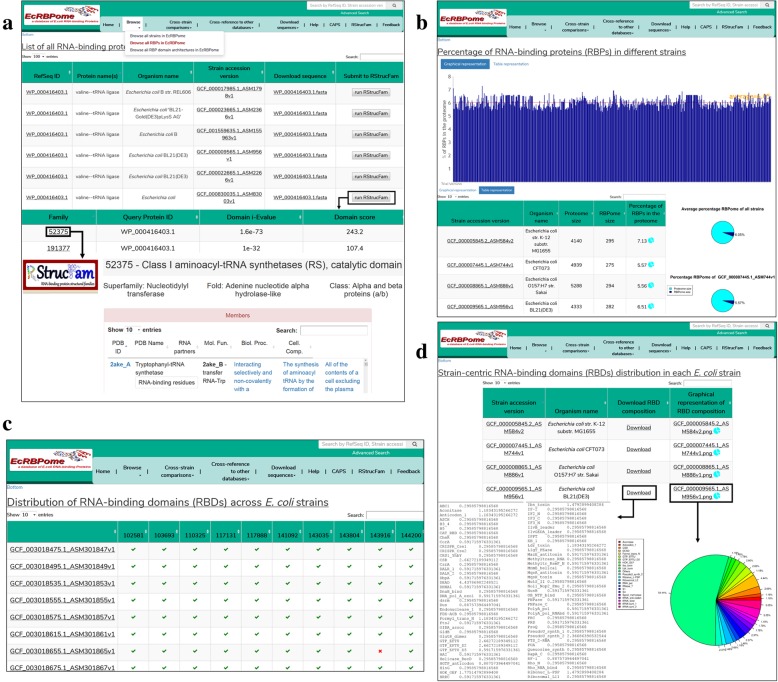
Database organisation and features. The organisation of the EcRBPome database and its important features have been represented in this figure. **a**. Sequence submission to RStrucFam, for the prediction of putative function(s) and cognate RNA partners. The snippets show the results page and the navigation to the RStrucFam web server for the details of the identified family(ies) have also been depicted. **b**. Graphical and tabular representations of the percentage of RBPs in the strains present in this database. Comparative pie-charts for these values in each strain and the average across all strains, are available for user download. **c**. Matrix representations for the distributions of various RBDs across the different *E. coli* strains. Presence of a particular RBD in a strain is denoted with a green tick mark, whereas absence is denoted by a red cross mark. **d**. RBD composition of each strain are available as user downloadable pie charts, as well as tab separated text files

The sequences of the RBPs can also be submitted to RStrucFam [12], for the prediction of their function and cognate RNA partner(s). Figure 1a demonstrates sequence submission to RStrucFam (from the ‘Browse all RBPs in EcRBPome’ option, under the ‘Browse’ menu), followed by the display of results, and navigation to the RStrucFam web server for the details of the identified family(ies). The RStrucFam can further be useful to search RBPs in the input sequence(s) or even entire bacterial proteomes. The RStrucFam server takes less than 3 minutes to search a typical bacterial proteome of around 5000 sequences.

#### Cross-strain comparisons

The various *E. coli* strains present in this database are compared on the basis of different parameters like, percentage of RBPs in each proteome (downloadable graphical representations, as well as comparative account with the average RBP percentage across all strains) (Fig. 1b), presence or absence of RBDs in each strain (matrix representation) (Fig. 1c), as well as percentage of the various RBDs in each strain (graphical representations and downloadable tab separated text files) (Fig. 1d). A pairwise comparison of two strains based on the presence of RBDs can be carried out. The RBPs obtained from 614 different *E. coli* strains were compared in terms of sequence, on the basis of single-link clustering method (please see Additional file 1 for a description of the method).

#### Cross-reference to other databases

EcRBPome provides annotations for each RBP by establishing links to other resources like, UniProt [13] (sequence annotation database), Protein Data Bank (PDB) [14] (structure annotation database) and Gene Ontology (GO) [15] and Enzyme Commissions (functional annotation resources).

#### Download sequences

FASTA sequences of RBPs encoded in each strain, all RBPs present in this database and those of RBDs predicted to be encoded in these RBPs are available for download by the users.

Further details of the features have been made available in the database ‘Help’ page and also as a help video (Additional file 2).


Additional file 2:Supplementary Video. Various features of the database have been presented in this file (MP4 11774 kb)


## Utility and discussion

To the best of our knowledge, EcRBPome is the first database of its kind that organises all RBPs known in a model organism in one platform. EcRBPome records information from all known complete *E. coli* proteomes (as of October 2018), and also links the data present in this database to other sequence, structure and function annotation resources. Hence, it is a ‘one-stop solution’ for all researchers who prefer to understand the global landscape of *E. coli* RBPs, as well as those who are interested in specific strains or proteins. It also predicts the function(s) and cognate RNA partner(s) for each of the RBPs present in this database, through our in-house algorithm, named RStrucFam. A total of 419 gene products, annotated as ‘hypothetical protein’ could be assigned to one of the RBP families (Additional file 3:  Table S1).

In addition, many other gene products (2007 RBPs), with a previously annotated primary function, have been predicted to retain RNA-binding property through our pipeline and mathematical models. For example, RStrucFam [12] and EcRBPome, could identify ‘moonlighting’ RNA-binding property in a protein of interest (riboflavin biosynthesis protein, RibD). This query sequence, with RefSeq ID: WP_001150457.1, is annotated as a bifunctional diaminohydroxyphosphoribosylaminopyrimidine deaminase/5-amino-6-(5 phosphoribosylamino) uracil reductase’) and is conserved in 149 out of the 614 strains recorded in EcRBPome. The protein associates with two UniProt entries (IDs: P25539 and Q3ZUB0), and three PDB structures (codes: 2G6V, 2O7P and 2OBC [16]) and none of these connections had earlier suggested RNA-binding function. The query sequence was predicted to associate with a ‘populated SCOP family’ (ID: 89800) associated with a single-membered PDB chain (ID: 2B3JD; RNA partner chain IDs: 2B3JE, 2B3JF and 2B3JH) through RStrucFam. Hence, RStrucFam predicted that the query protein can also bind to these aforementioned RNA chains, which are redundant in terms of sequence. It should be noted that there were no previous literature reports that associated an RNA-binding property with the RibD protein.

**Fig. 2:**
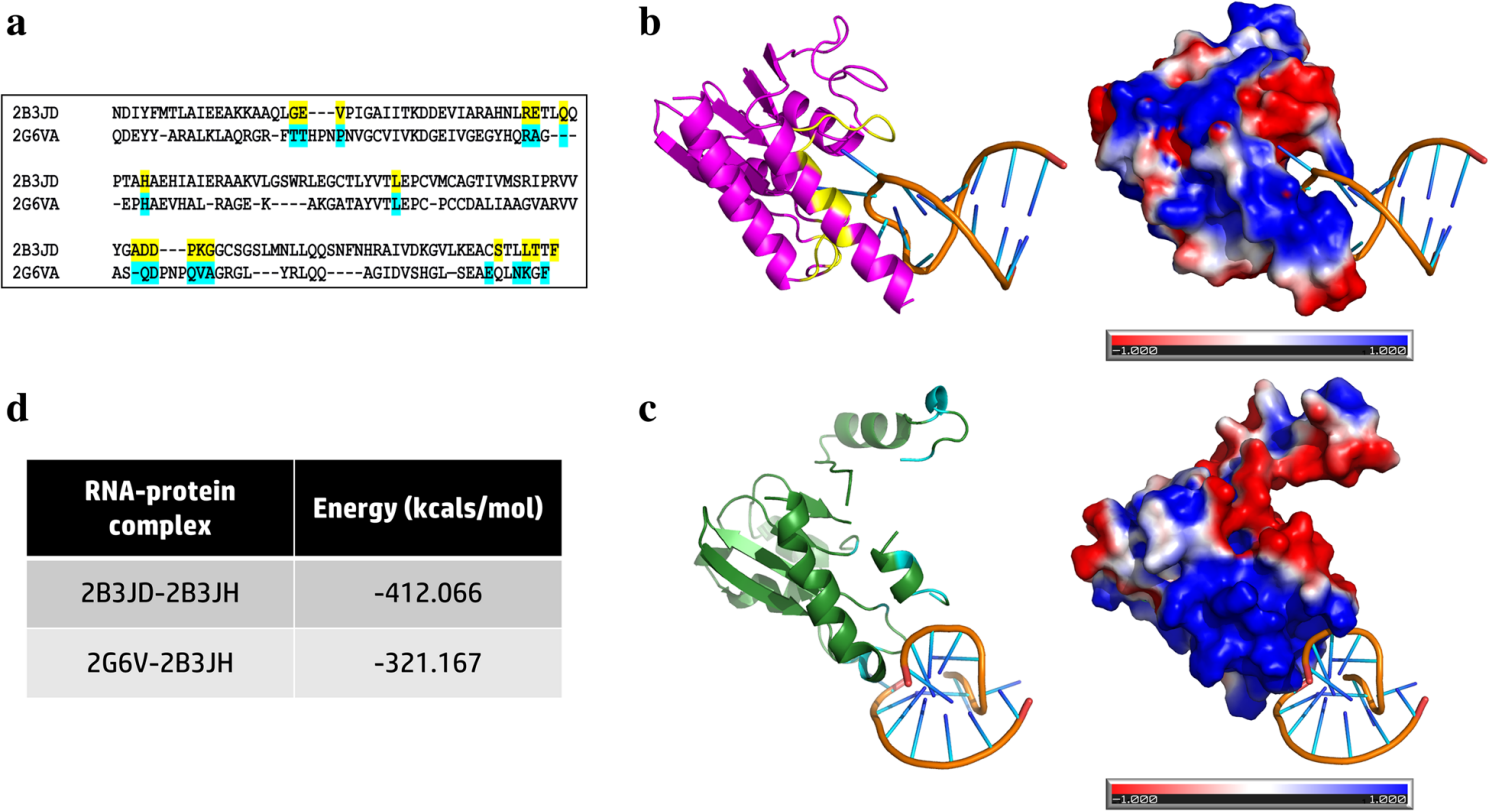
Comparison of RNA-binding affinities of two proteins. The RNA-binding properties of two proteins have been compared in this case study, on the basis of predictions made by RStrucFam. **a**. Structural alignment of the two proteins. The RNA-binding residues in 2B3JD (on the basis of 5 Å distance cut-off criterion) have been highlighted in yellow, whereas the structurally aligned residues in 2G6VA have been highlighted in cyan. The same colour scheme have also been followed in panels B and C of this figure. **b**. Structure of the 2B3JD-2B3JH complex (left pane) and its electrostatics properties on the solvent accessible surface (right pane). **c**. Structure of the 2G6VA-2B3JH complex (left pane) and its electrostatics properties on the solvent accessible surface (right pane). **d**. The potential energies of the two complexes (in kcals/mol) have been tabulated. These values were calculated using SYBYL7.2 (Force Field: Tripos, Electrostatics: None) in vacuum, post energy minimisations until convergence

Structural alignment of 2B3JD and largest of the query protein structures, 2G6VA (with the best resolution) were performed using the structural alignment tool, Matt [17]. The RNA-interacting residues in 2B3JD, as predicted by the RStrucFam algorithm, using 5 Å distance cut-off criterion, have been highlighted in yellow in Fig. 2a. The residues in 2G6VA that are structurally aligned with the above-mentioned residues, have been highlighted in cyan in Fig. 2a. Further, these equivalent residues were used to guide the docking of the RNA chain (2B3JH) onto the protein chain (2G6VA), using the docking tool HADDOCK [18]. The structures of the RNA-protein complexes (2B3JD-2B3JH and 2G6VA-2B3JH) have been shown on the left panes of Fig. 2b and c, respectively. The colour coding used to highlight the residues are same as those followed in Fig. 2a.

Electrostatic potential w*as* calculated using PDB2PQR [19] (in the AMBER force field) and Adaptive Poisson-Boltzmann Solver (APBS) [19, 20]. The ±1 kT/e (where, ‘k’ is the Boltzmann’s constant, ‘T’ is temperature in Kelvin and ‘e’ is the charge of an electron) electrostatic potential on the solvent accessible surfaces of the proteins have been shown on the right panes of Fig. 2b and c, for the 2B3JD-2B3JH and query bound to RNA, respectively. It is to be noted that in both the cases, the partner RNA binds amidst a large electropositive patch. These complexes were subjected to energy minimisations until convergence using SYBYL7.2 (Force Field: Tripos, Electrostatics: None) in vacuum and their potential energy values have been represented in Fig. 2d. This proves that proteins belonging to the same structural family are capable of binding to the same RNA, but perhaps with differential RNA-binding affinities, as seen in our previous studies also [21].

Interestingly, none of these residues are associated with the originally annotated bifunctional enzymatic activity. On the analysis of the NADP-bound structure of this protein (PDB code: 2O7P), it was seen that RibD uses a different site to bind the oxidised NADP^+^ cofactor, which does not overlap with the RNA-binding site that we have proposed here. Similarly, EcRBPome can be used in conjunction with RStrucFam to understand the RNA-binding properties of many uncharacterised proteins and so-called ‘non-RBPs’ in *E. coli* (with moonlighting RNA-binding properties), which might be of special interest to researchers working with the molecular biology of the *E. coli* model system. These moonlighting RBPs cannot be identified by pure sequence search-based methods, like BLAST, due to the lack of structural restraints in these searches.

With the growing advent of next generation sequencing technologies, the gap between protein sequence data and their functional annotation is ever-increasing. Biochemical functional tests can assign a ‘dominant’ (primary) function to these proteins but fail to foresee the ‘recessive’ (secondary) function. Due to the immense importance of RBPs in molecular processes, it is important to identify all RBPs (with RNA-binding as a primary or secondary function), which might help the biological fraternity to address many unanswered questions. On these lines, EcRBPome will serve as a reference to all RBPs in the *E. coli* model system. Homology-based inferences maybe further drawn from *E. coli* to assign RNA-binding properties to yet-unknown ‘RBPs’ in higher organisms, including humans.

## Conclusions

RBPs and sRNAs play important roles in bacterial post-transcriptional regulation of gene expression, and have been highly studied over the past decade [22, 23]. The number of complete genome sequences available has exponentially increased due to the advent of next generation sequencing technologies. Detailed structural and functional characterisation of several RBPs, even within *E. coli* genome, requires painstaking efforts and huge amounts of time. Computational approaches offer the first glimpse of putative RBPs using mathematical models of known RBPs and searches in whole genomes.

EcRBPome is a comprehensive platform for information on all RBPs from a popular model organism, *E. coli*. Sequences of RBPs reported in this database can also be used to select target gene products for detailed characterisation and to serve as start points for identifying sequence homologues in other microbial proteomes. Especially, the less studied species, where performing studies using experimental techniques are a challenge. For example, gene products of microorganisms that are highly pathogenic or the ones that are difficult to culture in the laboratory could be studied using this approach. The existing study will be further extended to the ever-growing number of complete *E. coli* proteomes and the EcRBPome will be updated with cross-references to a greater number of in-house, as well as external databases and softwares, to enrich the existing repository of information. RBPs can then be followed over taxonomic lineages to understand their patterns of conservation.

## Additional files

Additional file 1: Supplementary Methods. Further details of the genome-wide survey and cross-genome comparison methods have been presented in this file (DOCX 21 kb)

Additional file 3: **Table S1v** List of hypothetical proteins from *E. coli* proteomes that were annotated as RNA binding proteins through detection of RNA binding domain (DOCX 49 kb)

## Abbreviations

DA: Domain architecture
*E. coli*: *Escherichia coli*
GWS: Genome-wide survey
PDB: Protein Data Bank
RBD: RNA-binding domain
RBP: RNA-binding protein
sRNA: Small RNA
