# Supplementary material for: EcRBPome: a comprehensive database of all known E. coli RNA-binding proteins
Source: BMC Genomics. 2019 May 22;20:403. doi: 10.1186/s12864-019-5755-5 (PMC6530084; doi:10.1186/s12864-019-5755-5)
Supplement: Supplementary file 2 — Supplementary Methods. Further details of the genome-wide survey and cross-genome comparison methods have been presented in this file (DOCX 21 kb) [file 12864_2019_5755_MOESM1_ESM.docx]

**Additional file 1**

**Search method**

The search method was described in our previous study [1]. The start-points for this method were already reported sequence and structure signatures of RBPs, organised as structure-centric and sequence-centric family Hidden Markov Models (HMMs). 437 structure-centric RBP families were obtained using structure-based sequence alignments of known RBP structures, deposited in the PDB in complex with RNA. 746 sequence-centric RBP families were retrieved from the Pfam 28 database, on the basis of keyword search. Combined together, a total of 1183 RBP family HMMs were used to survey 614 *E. coli* proteomes for proteins with RNA-binding signatures. A sequence E-value cut-off of 10^-3^ was used for genome-wide survey (GWS) of each *E. coli* proteome. The hits obtained were further filtered with a domain i-Evalue cut-off of 0.5, to identify a total of 11662 RBPs from 614 *E. coli* strains.­

**Single-link clustering method**

All the RBPs identified from 614 different strains of *E. coli*, were compared by performing all-against-all protein sequence homology searches using the BLASTP module of the NCBI BLAST 2.2.30+ suite [2] with a sequence E-value cut-off of 10^-5^. The hits were clustered on the basis of 30% sequence identity and 70% query coverage cut-offs to identify *similar* proteins i.e., proteins that had a sequence identity of greater than or equal to 30%, as well as a query coverage of greater than or equal to 70%, were considered to homologous in terms of sequence and hence clustered. Pairs of proteins that share percentage identity and query coverage above the defined thresholds were first identified, followed by identification of common proteins (“links”) among the various protein pairs to define the clusters. The method has also been illustrated with a cartoon representation in the Help page of EcRBPome.

Clusters with a single member (without any identified homologues) have been called as single-member clusters (SMCs) and those with two or more members as multi-member clusters (MMCs).

# References

1. Ghosh P, Sowdhamini R (2016) Genome-wide survey of putative RNA-binding proteins encoded in the human proteome. *Mol BioSyst* **12**: 532–540.

2. Altschul SF, Gish W, Miller W, Myers EW, Lipman DJ (1990) Basic local alignment search tool. *J Mol Biol* **215**: 403–410.
