# Supplementary material for: EcRBPome: a comprehensive database of all known E. coli RNA-binding proteins
Source: BMC Genomics. 2019 May 22;20:403. doi: 10.1186/s12864-019-5755-5 (PMC6530084; doi:10.1186/s12864-019-5755-5)
Supplement: Supplementary file 3 — Table S1v List of hypothetical proteins from E. coli proteomes that were annotated as RNA binding proteins through detection of RNA binding domain (DOCX 49 kb) [file 12864_2019_5755_MOESM3_ESM.docx]

Additional file 3: **Table S1.** List of hypothetical proteins from E. coli proteomes that were annotated as RNA binding proteins through detection of RNA binding domain derived from our sequence searches and recorded in EcRBPome. The RNA binding domain (RBD) and its position has been reported along with the number of strains.

| **ID of hypothetical protein** | **Number of strains** | **Number of RBDs** | **RBD (sequence position)** |
| --- | --- | --- | --- |
| NP_052606.1 | 1 | 1 | RnlA_toxin[90..312] |
| NP_308526.1 | 1 | 1 | SgrR_N[5..119] |
| NP_308561.1 | 1 | 1 | tRNA_edit[23..147] |
| NP_308647.1 | 1 | 1 | HOK_GEF[5..46] |
| NP_308648.1 | 1 | 1 | HOK_GEF[38..79] |
| NP_308826.2 | 1 | 1 | Aconitase[20..471] |
| NP_309027.1 | 1 | 1 | Trm112p[2..41] |
| NP_309283.1 | 1 | 1 | Ribonuc_L-PSP[10..126] |
| NP_309332.1 | 1 | 2 | DEAD[425..567];DNA_primase_S[141..253] |
| NP_309373.1 | 1 | 1 | PELOTA_1[160..242] |
| NP_309866.4 | 1 | 1 | Sua5_yciO_yrdC[22..198] |
| NP_310523.1 | 1 | 1 | tRNA_edit[30..155] |
| NP_310545.2 | 1 | 1 | Ribonuc_L-PSP[2..114] |
| NP_311363.1 | 1 | 1 | Helicase_RecD[196..337] |
| NP_311507.2 | 1 | 1 | Ub-RnfH[5..87] |
| NP_311637.2 | 1 | 1 | 231597[45..258] |
| NP_311638.1 | 1 | 1 | Cluster121[1..186] |
| NP_311639.1 | 1 | 1 | Cluster26[1..345] |
| NP_311640.1 | 1 | 1 | CRISPR_Cse2[25..167] |
| NP_311641.1 | 1 | 1 | CRISPR_Cse1[5..472] |
| NP_311643.1 | 1 | 1 | HOK_GEF[5..46] |
| NP_311799.1 | 1 | 1 | ASCH[6..103] |
| NP_311983.1 | 1 | 1 | tRNA_bind[14..108] |
| NP_312020.2 | 1 | 1 | Ribonuc_L-PSP[7..127] |
| NP_312276.2 | 1 | 1 | S1[647..720] |
| NP_312291.1 | 1 | 1 | RtcB[11..408] |
| NP_312398.1 | 1 | 1 | RsmJ[33..278] |
| NP_313170.1 | 1 | 1 | YjeF_N[38..199] |
| NP_313247.1 | 1 | 1 | Ribonuc_L-PSP[20..140] |
| NP_313252.1 | 1 | 1 | Ribonuc_L-PSP[11..128] |
| NP_313287.1 | 1 | 1 | DEAD[94..300] |
| NP_944505.1 | 1 | 1 | ASCH[16..144] |
| NP_944516.1 | 1 | 1 | HOK_GEF[5..44] |
| NP_944589.1 | 1 | 1 | HOK_GEF[25..66] |
| WP_000066189.1 | 2 | 1 | KilA-N[12..104] |
| WP_000132601.1 | 77 | 1 | SymE_toxin[20..71] |
| WP_000132602.1 | 2 | 1 | SymE_toxin[20..71] |
| WP_000132612.1 | 25 | 1 | SymE_toxin[20..71] |
| WP_000132614.1 | 11 | 1 | SymE_toxin[20..71] |
| WP_000132616.1 | 1 | 1 | SymE_toxin[20..71] |
| WP_000132621.1 | 22 | 1 | SymE_toxin[20..71] |
| WP_000132625.1 | 2 | 1 | SymE_toxin[20..71] |
| WP_000132630.1 | 56 | 1 | SymE_toxin[20..71] |
| WP_000132631.1 | 7 | 1 | SymE_toxin[20..71] |
| WP_000132632.1 | 3 | 1 | SymE_toxin[20..71] |
| WP_000132633.1 | 4 | 1 | SymE_toxin[20..71] |
| WP_000135003.1 | 1 | 1 | YafO_toxin[24..124] |
| WP_000158029.1 | 9 | 1 | Ribonuc_L-PSP[2..114] |
| WP_000163465.1 | 1 | 1 | MT-A70[5..176] |
| WP_000164226.1 | 1 | 1 | ProQ[64..174] |
| WP_000164228.1 | 5 | 1 | ProQ[60..171] |
| WP_000166947.1 | 1 | 1 | ProQ[5..114] |
| WP_000166948.1 | 3 | 1 | ProQ[5..114] |
| WP_000166949.1 | 2 | 1 | ProQ[5..114] |
| WP_000166950.1 | 1 | 1 | ProQ[5..114] |
| WP_000166952.1 | 7 | 1 | ProQ[5..114] |
| WP_000170743.1 | 5 | 1 | Ldr_toxin[1..35] |
| WP_000170745.1 | 27 | 1 | Ldr_toxin[1..35] |
| WP_000170748.1 | 3 | 1 | Ldr_toxin[1..35] |
| WP_000170926.1 | 40 | 1 | Ldr_toxin[1..35] |
| WP_000170951.1 | 7 | 1 | Ldr_toxin[1..35] |
| WP_000170954.1 | 242 | 1 | Ldr_toxin[1..35] |
| WP_000170956.1 | 21 | 1 | Ldr_toxin[1..35] |
| WP_000170957.1 | 6 | 1 | Ldr_toxin[1..35] |
| WP_000170965.1 | 176 | 1 | Ldr_toxin[1..35] |
| WP_000170970.1 | 2 | 1 | Ldr_toxin[1..35] |
| WP_000170971.1 | 5 | 1 | Ldr_toxin[1..35] |
| WP_000170972.1 | 4 | 1 | Ldr_toxin[1..35] |
| WP_000176713.1 | 23 | 1 | Ldr_toxin[1..35] |
| WP_000178825.1 | 1 | 1 | KilA-N[12..102] |
| WP_000191544.1 | 2 | 1 | KilA-N[11..113] |
| WP_000200358.1 | 11 | 1 | KilA-N[11..113] |
| WP_000209995.1 | 2 | 1 | ProQ[65..175] |
| WP_000230262.1 | 14 | 1 | Ribonuc_L-PSP[11..128] |
| WP_000230281.1 | 282 | 1 | Ribonuc_L-PSP[11..128] |
| WP_000250467.1 | 1 | 1 | YafO_toxin[30..147] |
| WP_000258197.1 | 2 | 1 | ProQ[50..158] |
| WP_000258198.1 | 4 | 1 | ProQ[50..158] |
| WP_000258199.1 | 4 | 1 | ProQ[50..158] |
| WP_000361403.1 | 1 | 1 | Viral_helicase1[121..331] |
| WP_000387271.1 | 2 | 1 | MT-A70[7..180] |
| WP_000415585.1 | 16 | 1 | MqsR_toxin[5..97] |
| WP_000424040.1 | 6 | 1 | DEAD[9..153] |
| WP_000431541.1 | 7 | 1 | YafO_toxin[25..126] |
| WP_000439434.1 | 1 | 1 | 82075[1..110] |
| WP_000479990.1 | 5 | 1 | YafO_toxin[30..146] |
| WP_000515888.1 | 1 | 1 | ProQ[82..196] |
| WP_000515889.1 | 1 | 1 | ProQ[83..197] |
| WP_000517686.1 | 2 | 1 | ProQ[81..195] |
| WP_000517687.1 | 7 | 1 | ProQ[81..195] |
| WP_000517688.1 | 1 | 1 | ProQ[81..195] |
| WP_000517689.1 | 8 | 1 | ProQ[81..195] |
| WP_000517692.1 | 5 | 1 | ProQ[81..195] |
| WP_000517694.1 | 8 | 1 | ProQ[81..195] |
| WP_000517695.1 | 11 | 1 | ProQ[81..195] |
| WP_000517696.1 | 3 | 1 | ProQ[81..195] |
| WP_000517697.1 | 3 | 1 | ProQ[81..195] |
| WP_000521602.1 | 2 | 1 | ProQ[82..195] |
| WP_000521603.1 | 20 | 1 | ProQ[82..194] |
| WP_000521604.1 | 6 | 1 | ProQ[82..195] |
| WP_000521606.1 | 3 | 1 | ProQ[81..195] |
| WP_000536047.1 | 5 | 1 | ProQ[82..193] |
| WP_000537018.1 | 2 | 1 | ProQ[63..172] |
| WP_000549966.1 | 3 | 1 | YafO_toxin[35..142] |
| WP_000686652.1 | 4 | 1 | Viral_helicase1[268..493] |
| WP_000768220.1 | 5 | 1 | DEAD[81..263] |
| WP_000866321.1 | 27 | 1 | ASCH[5..99] |
| WP_000877024.1 | 7 | 1 | KilA-N[5..116] |
| WP_000932553.1 | 2 | 1 | YafO_toxin[29..145] |
| WP_000934358.1 | 2 | 1 | RVT_1[63..251] |
| WP_000950181.1 | 42 | 1 | Ldr_toxin[10..44] |
| WP_000950187.1 | 30 | 1 | Ldr_toxin[10..44] |
| WP_001037485.1 | 1 | 1 | Viral_helicase1[107..366] |
| WP_001046389.1 | 1 | 1 | MT-A70[4..175] |
| WP_001060285.1 | 1 | 1 | KilA-N[12..104] |
| WP_001061378.1 | 4 | 1 | KilA-N[4..111] |
| WP_001061379.1 | 4 | 1 | KilA-N[4..111] |
| WP_001061380.1 | 6 | 1 | KilA-N[4..111] |
| WP_001061385.1 | 1 | 1 | KilA-N[4..111] |
| WP_001061386.1 | 4 | 1 | KilA-N[4..106] |
| WP_001061397.1 | 7 | 1 | KilA-N[4..107] |
| WP_001061398.1 | 3 | 1 | KilA-N[4..107] |
| WP_001061403.1 | 4 | 1 | KilA-N[4..107] |
| WP_001061404.1 | 11 | 1 | KilA-N[4..107] |
| WP_001061405.1 | 1 | 1 | KilA-N[4..107] |
| WP_001061408.1 | 9 | 1 | KilA-N[4..107] |
| WP_001061410.1 | 1 | 1 | KilA-N[4..107] |
| WP_001061411.1 | 18 | 1 | KilA-N[4..107] |
| WP_001061412.1 | 1 | 1 | KilA-N[4..107] |
| WP_001061413.1 | 14 | 1 | KilA-N[4..107] |
| WP_001061416.1 | 2 | 1 | KilA-N[4..107] |
| WP_001061417.1 | 2 | 1 | KilA-N[4..107] |
| WP_001061422.1 | 7 | 1 | KilA-N[4..122] |
| WP_001061427.1 | 8 | 1 | KilA-N[4..122] |
| WP_001061438.1 | 19 | 1 | KilA-N[4..111] |
| WP_001061441.1 | 2 | 1 | KilA-N[4..111] |
| WP_001061444.1 | 27 | 1 | KilA-N[4..111] |
| WP_001061445.1 | 8 | 1 | KilA-N[4..111] |
| WP_001061449.1 | 2 | 1 | KilA-N[4..111] |
| WP_001072669.1 | 19 | 1 | KilA-N[5..115] |
| WP_001072670.1 | 1 | 1 | KilA-N[5..115] |
| WP_001072672.1 | 4 | 1 | KilA-N[5..115] |
| WP_001072673.1 | 7 | 1 | KilA-N[5..115] |
| WP_001076065.1 | 1 | 1 | ProQ[28..137] |
| WP_001113989.1 | 121 | 1 | zf-FPG_IleRS[235..263] |
| WP_001114025.1 | 90 | 1 | zf-FPG_IleRS[235..263] |
| WP_001114026.1 | 67 | 1 | zf-FPG_IleRS[235..263] |
| WP_001114035.1 | 18 | 1 | zf-FPG_IleRS[235..263] |
| WP_001135718.1 | 3 | 1 | HOK_GEF[5..44] |
| WP_001182410.1 | 10 | 2 | PELOTA_1[275..357];PRTase_1[9..249] |
| WP_001182411.1 | 7 | 2 | PELOTA_1[275..357];PRTase_1[9..249] |
| WP_001182418.1 | 97 | 2 | PELOTA_1[275..357];PRTase_1[9..249] |
| WP_001182419.1 | 2 | 2 | PELOTA_1[275..357];PRTase_1[9..249] |
| WP_001182420.1 | 2 | 2 | PELOTA_1[275..357];PRTase_1[9..249] |
| WP_001182421.1 | 1 | 2 | PELOTA_1[275..357];PRTase_1[9..249] |
| WP_001198454.1 | 7 | 1 | YafO_toxin[35..145] |
| WP_001238187.1 | 1 | 1 | SgrR_N[5..119] |
| WP_001238188.1 | 5 | 1 | SgrR_N[5..119] |
| WP_001238189.1 | 6 | 1 | SgrR_N[5..119] |
| WP_001238190.1 | 11 | 1 | SgrR_N[5..119] |
| WP_001238194.1 | 17 | 1 | SgrR_N[5..119] |
| WP_001238196.1 | 2 | 1 | SgrR_N[5..119] |
| WP_001238197.1 | 1 | 1 | SgrR_N[5..119] |
| WP_001238199.1 | 1 | 1 | SgrR_N[5..119] |
| WP_001238202.1 | 41 | 1 | SgrR_N[5..119] |
| WP_001238207.1 | 7 | 1 | SgrR_N[5..119] |
| WP_001238217.1 | 8 | 1 | SgrR_N[5..119] |
| WP_001238218.1 | 7 | 1 | SgrR_N[5..119] |
| WP_001238224.1 | 31 | 1 | SgrR_N[5..119] |
| WP_001238226.1 | 17 | 1 | SgrR_N[5..119] |
| WP_001238227.1 | 1 | 1 | SgrR_N[5..119] |
| WP_001238231.1 | 88 | 1 | SgrR_N[5..119] |
| WP_001238232.1 | 14 | 1 | SgrR_N[5..119] |
| WP_001238233.1 | 4 | 1 | SgrR_N[5..119] |
| WP_001238237.1 | 13 | 1 | SgrR_N[5..119] |
| WP_001238238.1 | 10 | 1 | SgrR_N[5..119] |
| WP_001238241.1 | 25 | 1 | SgrR_N[5..119] |
| WP_001238242.1 | 4 | 1 | SgrR_N[5..119] |
| WP_001238243.1 | 1 | 1 | SgrR_N[5..119] |
| WP_001238246.1 | 4 | 1 | SgrR_N[5..119] |
| WP_001238248.1 | 8 | 1 | SgrR_N[5..119] |
| WP_001238251.1 | 2 | 1 | SgrR_N[5..119] |
| WP_001238254.1 | 1 | 1 | SgrR_N[5..119] |
| WP_001238258.1 | 43 | 1 | SgrR_N[5..119] |
| WP_001238259.1 | 9 | 1 | SgrR_N[5..119] |
| WP_001238260.1 | 3 | 1 | SgrR_N[5..119] |
| WP_001238261.1 | 1 | 1 | SgrR_N[5..119] |
| WP_001278274.1 | 1 | 1 | GIIM[76..167] |
| WP_001295493.1 | 548 | 1 | Ribonuc_L-PSP[2..114] |
| WP_001310439.1 | 4 | 1 | ProQ[76..187] |
| WP_001350482.1 | 45 | 1 | RVT_1[91..337] |
| WP_001351125.1 | 23 | 1 | Ribonuc_L-PSP[2..114] |
| WP_001356710.1 | 21 | 1 | ProQ[76..187] |
| WP_001357525.1 | 3 | 1 | SymE_toxin[20..71] |
| WP_001400834.1 | 5 | 1 | Ldr_toxin[1..35] |
| WP_001401193.1 | 7 | 1 | Ldr_toxin[1..35] |
| WP_001401779.1 | 1 | 1 | ProQ[81..195] |
| WP_001415809.1 | 1 | 1 | ProQ[76..187] |
| WP_001441942.1 | 2 | 1 | Ldr_toxin[1..35] |
| WP_001522303.1 | 3 | 1 | ProQ[50..158] |
| WP_001525367.1 | 1 | 1 | MT-A70[5..176] |
| WP_001527562.1 | 1 | 1 | RNA_ligase[37..205] |
| WP_001536522.1 | 1 | 1 | ProQ[50..159] |
| WP_001544814.1 | 1 | 1 | YafO_toxin[44..161] |
| WP_001544870.1 | 1 | 2 | CwfJ_C_1[24..139];DcpS_C[23..135] |
| WP_001545777.1 | 1 | 1 | Viral_helicase1[269..492] |
| WP_001549206.1 | 1 | 1 | DEAD[60..225] |
| WP_001555163.1 | 2 | 1 | YafO_toxin[30..146] |
| WP_001555612.1 | 1 | 1 | YafO_toxin[44..161] |
| WP_001561911.1 | 1 | 1 | RNA_ligase[37..205] |
| WP_001565131.1 | 1 | 1 | ProQ[60..172] |
| WP_001566978.1 | 1 | 1 | ProQ[5..114] |
| WP_001568072.1 | 1 | 2 | FinO_N[1..70];ProQ[83..192] |
| WP_001570696.1 | 1 | 1 | 82075[1..108] |
| WP_001585166.1 | 1 | 2 | PELOTA_1[275..357];PRTase_1[9..249] |
| WP_001589603.1 | 4 | 1 | SymE_toxin[20..71] |
| WP_001595233.1 | 1 | 1 | ProQ[81..196] |
| WP_001603073.1 | 6 | 1 | PIN[8..119] |
| WP_001610752.1 | 3 | 1 | DEAD[25..202] |
| WP_001613653.1 | 5 | 1 | RNA_ligase[37..205] |
| WP_001617199.1 | 2 | 1 | MT-A70[5..176] |
| WP_001683189.1 | 1 | 1 | DEAD[105..230] |
| WP_001683215.1 | 1 | 1 | RNA_ligase[37..205] |
| WP_001683557.1 | 1 | 1 | Viral_helicase1[23..248] |
| WP_001703912.1 | 14 | 1 | ProQ[81..195] |
| WP_004099044.1 | 1 | 1 | Ribonuc_L-PSP[13..132] |
| WP_004152388.1 | 3 | 1 | ProQ[64..177] |
| WP_004210251.1 | 1 | 2 | PELOTA_1[275..357];PRTase_1[9..249] |
| WP_011091027.1 | 2 | 1 | 82075[1..110] |
| WP_014639208.1 | 10 | 1 | Ribonuc_L-PSP[2..114] |
| WP_015967850.1 | 5 | 1 | KilA-N[4..111] |
| WP_016236507.1 | 1 | 1 | tRNA_bind[15..109] |
| WP_016240611.1 | 3 | 1 | DEAD[308..476] |
| WP_016244154.1 | 3 | 1 | ProQ[50..159] |
| WP_016809719.1 | 2 | 1 | DEAD[148..308] |
| WP_021292868.1 | 2 | 1 | SgrR_N[5..119] |
| WP_021513175.1 | 2 | 1 | DEAD[15..191] |
| WP_021522449.1 | 1 | 1 | ProQ[5..113] |
| WP_021530973.1 | 1 | 1 | DEAD[36..208] |
| WP_021536204.1 | 1 | 1 | ProQ[83..194] |
| WP_021543045.1 | 1 | 1 | ProQ[60..165] |
| WP_021543214.1 | 1 | 1 | KilA-N[3..102] |
| WP_021557482.1 | 1 | 1 | DEAD[40..206] |
| WP_021560253.1 | 1 | 1 | MT-A70[4..175] |
| WP_021560741.1 | 1 | 1 | MT-A70[4..175] |
| WP_021561689.1 | 1 | 1 | ProQ[81..195] |
| WP_021572006.1 | 2 | 1 | ProQ[50..159] |
| WP_021578776.1 | 2 | 1 | Colicin_immun[1..86] |
| WP_021580444.1 | 1 | 1 | DEAD[40..206] |
| WP_022296622.1 | 1 | 1 | SgrR_N[5..119] |
| WP_023147588.1 | 6 | 1 | ProQ[1..82] |
| WP_023278667.1 | 1 | 1 | MT-A70[7..180] |
| WP_023565512.1 | 1 | 1 | YafO_toxin[24..123] |
| WP_023908880.1 | 3 | 1 | KilA-N[4..111] |
| WP_024171396.1 | 3 | 1 | ProQ[68..180] |
| WP_024174015.1 | 2 | 1 | Colicin[236..421] |
| WP_024174336.1 | 1 | 1 | Ibs_toxin[77..95] |
| WP_024191425.1 | 1 | 1 | ProQ[82..194] |
| WP_024215468.1 | 1 | 1 | SgrR_N[5..119] |
| WP_024218636.1 | 1 | 1 | Ribonuc_L-PSP[2..114] |
| WP_024219727.1 | 1 | 1 | KilA-N[4..111] |
| WP_024220650.1 | 6 | 1 | KilA-N[4..111] |
| WP_024223152.1 | 1 | 1 | SgrR_N[5..119] |
| WP_024227171.1 | 1 | 1 | Ribonuc_L-PSP[2..114] |
| WP_024227971.1 | 8 | 1 | KilA-N[4..111] |
| WP_024243782.1 | 1 | 1 | Ribonuc_L-PSP[2..114] |
| WP_024946528.1 | 4 | 1 | YafO_toxin[29..145] |
| WP_025263476.1 | 1 | 1 | ProQ[81..195] |
| WP_025380699.1 | 2 | 1 | KilA-N[4..107] |
| WP_025750220.1 | 1 | 1 | ProQ[82..195] |
| WP_029488747.1 | 1 | 1 | KilA-N[4..111] |
| WP_032178322.1 | 2 | 1 | SgrR_N[5..119] |
| WP_032178584.1 | 1 | 1 | KilA-N[5..116] |
| WP_032200873.1 | 4 | 1 | SgrR_N[5..119] |
| WP_032208371.1 | 2 | 1 | SgrR_N[5..119] |
| WP_032211321.1 | 1 | 1 | RPAP2_Rtr1[3..57] |
| WP_032314631.1 | 2 | 1 | SgrR_N[5..119] |
| WP_032610457.1 | 2 | 2 | PELOTA_1[275..357];PRTase_1[9..249] |
| WP_033552788.1 | 1 | 1 | B3_4[62..210] |
| WP_033816113.1 | 1 | 1 | SgrR_N[5..119] |
| WP_038813125.1 | 2 | 1 | ProQ[8..115] |
| WP_039066042.1 | 1 | 1 | Ribonuc_L-PSP[2..114] |
| WP_039268320.1 | 1 | 1 | SgrR_N[5..119] |
| WP_040116959.1 | 1 | 1 | 82075[1..110] |
| WP_042634304.1 | 11 | 2 | PELOTA_1[275..357];PRTase_1[9..249] |
| WP_044311195.1 | 1 | 1 | DEAD[36..170] |
| WP_046881598.1 | 1 | 1 | Viral_helicase1[270..498] |
| WP_047675865.1 | 1 | 1 | SgrR_N[5..119] |
| WP_049590554.1 | 6 | 2 | PELOTA_1[275..357];PRTase_1[9..249] |
| WP_053264879.1 | 2 | 1 | SgrR_N[5..119] |
| WP_057696394.1 | 1 | 1 | SgrR_N[5..119] |
| WP_059331900.1 | 1 | 1 | YafO_toxin[29..145] |
| WP_062946177.1 | 1 | 1 | SgrR_N[5..119] |
| WP_063073408.1 | 1 | 1 | Viral_helicase1[122..331] |
| WP_065226291.1 | 1 | 1 | SgrR_N[5..119] |
| WP_069684287.1 | 1 | 1 | SgrR_N[5..119] |
| WP_071524576.1 | 240 | 1 | Ibs_toxin[1..19] |
| WP_071525914.1 | 142 | 1 | Ibs_toxin[1..19] |
| WP_071776730.1 | 1 | 1 | RPAP2_Rtr1[1..65] |
| WP_071779209.1 | 2 | 1 | GIIM[3..81] |
| WP_072643897.1 | 1 | 1 | SgrR_N[5..119] |
| WP_073477109.1 | 1 | 1 | DEAD[15..191] |
| WP_073519757.1 | 3 | 1 | SgrR_N[5..119] |
| WP_073520811.1 | 2 | 1 | Colicin[223..405] |
| WP_073521327.1 | 1 | 1 | Viral_helicase1[270..492] |
| WP_073527453.1 | 1 | 1 | SgrR_N[5..119] |
| WP_073528121.1 | 1 | 1 | SgrR_N[5..119] |
| WP_073533401.1 | 1 | 1 | SgrR_N[5..119] |
| WP_073535451.1 | 1 | 1 | Colicin[223..405] |
| WP_073544919.1 | 1 | 1 | SgrR_N[5..119] |
| WP_077250878.1 | 3 | 1 | GIIM[1..75] |
| WP_077694932.1 | 1 | 1 | MT-A70[1..143] |
| WP_077766652.1 | 1 | 1 | DNA_primase_S[15..127] |
| WP_077781578.1 | 1 | 1 | SgrR_N[1..94] |
| WP_077897250.1 | 2 | 1 | DEAD[109..232] |
| WP_077898166.1 | 3 | 1 | Colicin[188..370] |
| WP_077898586.1 | 1 | 1 | Colicin[123..305] |
| WP_078163779.1 | 1 | 1 | tRNA_edit[1..102] |
| WP_078207734.1 | 1 | 2 | PELOTA_1[275..357];PRTase_1[9..249] |
| WP_080030292.1 | 1 | 1 | SgrR_N[5..119] |
| WP_086625076.1 | 2 | 1 | dsrm[142..208] |
| WP_094319298.1 | 1 | 1 | SgrR_N[5..119] |
| WP_096040739.1 | 1 | 1 | SgrR_N[5..119] |
| WP_096855513.1 | 1 | 1 | SgrR_N[5..119] |
| WP_097312644.1 | 1 | 1 | ProQ[81..192] |
| WP_098944884.1 | 1 | 1 | SgrR_N[5..119] |
| WP_099588340.1 | 1 | 1 | SgrR_N[5..119] |
| WP_100249717.1 | 8 | 1 | Ibs_toxin[1..19] |
| WP_100249771.1 | 20 | 1 | Ibs_toxin[1..18] |
| WP_100249772.1 | 38 | 1 | Ibs_toxin[1..18] |
| WP_100249843.1 | 2 | 1 | Ibs_toxin[1..18] |
| WP_100249846.1 | 50 | 1 | Ibs_toxin[1..19] |
| WP_100273061.1 | 1 | 1 | SgrR_N[5..119] |
| WP_100273070.1 | 6 | 1 | Ibs_toxin[1..18] |
| WP_100635201.1 | 9 | 1 | Ibs_toxin[1..19] |
| WP_100692412.1 | 4 | 1 | Ibs_toxin[1..18] |
| WP_100792650.1 | 8 | 1 | Ibs_toxin[1..18] |
| WP_101356890.1 | 1 | 1 | KilA-N[5..116] |
| WP_101356895.1 | 1 | 1 | YafO_toxin[35..145] |
| WP_101968853.1 | 1 | 1 | SgrR_N[5..119] |
| WP_101969345.1 | 1 | 1 | SgrR_N[5..119] |
| WP_101975665.1 | 3 | 1 | HOK_GEF[25..66] |
| WP_101979717.1 | 1 | 1 | HOK_GEF[25..66] |
| WP_101979777.1 | 1 | 1 | SgrR_N[5..119] |
| WP_104457503.1 | 1 | 1 | SgrR_N[5..119] |
| WP_106777931.1 | 6 | 1 | Ldr_toxin[72..102] |
| WP_106873672.1 | 1 | 1 | SgrR_N[5..119] |
| WP_106883996.1 | 2 | 1 | SgrR_N[5..119] |
| WP_106884706.1 | 1 | 1 | HOK_GEF[25..66] |
| WP_106888557.1 | 1 | 1 | tRNA_edit[30..155] |
| WP_106894701.1 | 1 | 1 | ProQ[82..193] |
| WP_106905916.1 | 1 | 1 | tRNA_edit[30..155] |
| WP_106906904.1 | 1 | 1 | Ribonuc_L-PSP[2..114] |
| WP_106907580.1 | 1 | 1 | SgrR_N[5..119] |
| WP_106907789.1 | 1 | 1 | Ibs_toxin[1..18] |
| WP_106910274.1 | 1 | 1 | HOK_GEF[25..66] |
| WP_111724575.1 | 1 | 1 | B3_4[62..213] |
| YP_002406272.1 | 1 | 1 | SgrR_N[5..119] |
| YP_002406401.1 | 1 | 1 | ASCH[27..144] |
| YP_002406713.1 | 1 | 1 | RtcB[33..465] |
| YP_002406765.1 | 1 | 1 | Aconitase[4..392] |
| YP_002407252.1 | 1 | 1 | Ribonuc_L-PSP[2..114] |
| YP_002407274.1 | 1 | 1 | tRNA_edit[30..155] |
| YP_002407603.1 | 1 | 1 | Sua5_yciO_yrdC[22..198] |
| YP_002408193.1 | 1 | 1 | Trm112p[2..41] |
| YP_002408758.1 | 1 | 1 | Ub-RnfH[5..87] |
| YP_002409238.1 | 1 | 1 | ASCH[6..103] |
| YP_002409476.1 | 1 | 1 | tRNA_bind[14..108] |
| YP_002409517.1 | 1 | 1 | Ribonuc_L-PSP[7..127] |
| YP_002409794.1 | 1 | 1 | RtcB[11..408] |
| YP_002410493.1 | 1 | 1 | YjeF_N[38..199] |
| YP_006118784.1 | 1 | 1 | SgrR_N[5..119] |
| YP_006118821.1 | 1 | 1 | tRNA_edit[23..147] |
| YP_006119049.1 | 1 | 1 | Aconitase[20..471] |
| YP_006119198.1 | 1 | 1 | Trm112p[2..41] |
| YP_006119340.1 | 1 | 1 | Ribonuc_L-PSP[10..126] |
| YP_006119607.1 | 1 | 1 | Ldr_toxin[10..44] |
| YP_006119662.1 | 1 | 1 | Sua5_yciO_yrdC[22..198] |
| YP_006120147.1 | 1 | 1 | tRNA_edit[30..155] |
| YP_006120166.1 | 1 | 1 | Ribonuc_L-PSP[3..115] |
| YP_006120351.1 | 1 | 1 | 82075[1..110] |
| YP_006120950.1 | 1 | 1 | Ub-RnfH[5..87] |
| YP_006121194.1 | 1 | 1 | ASCH[6..103] |
| YP_006121405.1 | 1 | 1 | tRNA_bind[14..108] |
| YP_006121440.1 | 1 | 1 | Ribonuc_L-PSP[7..127] |
| YP_006121729.1 | 1 | 1 | RtcB[11..408] |
| YP_006122577.1 | 1 | 1 | YjeF_N[38..199] |
| YP_006122658.1 | 1 | 1 | Ribonuc_L-PSP[7..127] |
| YP_006162210.1 | 1 | 1 | MazE_antitoxin[7..56] |
| YP_006162211.1 | 1 | 1 | PIN[5..131] |
| YP_006162216.1 | 1 | 1 | ProQ[82..194] |
| YP_006777122.1 | 1 | 1 | RtcB[11..408] |
| YP_006777713.1 | 1 | 1 | ASCH[6..103] |
| YP_006777858.1 | 1 | 1 | CRISPR_Cse1[5..472] |
| YP_006777859.1 | 1 | 1 | CRISPR_Cse2[25..167] |
| YP_006777860.1 | 1 | 1 | Cluster26[1..339] |
| YP_006777861.1 | 1 | 1 | Cluster121[1..182] |
| YP_006777862.1 | 1 | 1 | 231597[1..212] |
| YP_006777977.1 | 1 | 1 | Ub-RnfH[5..87] |
| YP_006778876.1 | 1 | 1 | Ribonuc_L-PSP[2..114] |
| YP_006778900.1 | 1 | 1 | tRNA_edit[30..155] |
| YP_006778976.1 | 1 | 1 | HOK_GEF[4..44] |
| YP_006779211.1 | 1 | 1 | HOK_GEF[5..44] |
| YP_006779489.1 | 1 | 1 | Sua5_yciO_yrdC[22..198] |
| YP_006779594.1 | 1 | 1 | Ldr_toxin[1..35] |
| YP_006779596.1 | 1 | 1 | Ldr_toxin[1..35] |
| YP_006779861.1 | 1 | 1 | Ribonuc_L-PSP[10..126] |
| YP_006779874.1 | 1 | 1 | HOK_GEF[5..44] |
| YP_006780027.1 | 1 | 1 | Trm112p[2..41] |
| YP_006780412.1 | 1 | 1 | HOK_GEF[5..46] |
| YP_006780488.1 | 1 | 1 | tRNA_edit[23..147] |
| YP_006780524.1 | 1 | 1 | SgrR_N[5..119] |
| YP_006780642.1 | 1 | 1 | ASCH[16..144] |
| YP_006780786.1 | 1 | 1 | RF-1[4..132] |
| YP_006781140.1 | 1 | 1 | Ribonuc_L-PSP[7..127] |
| YP_006781751.1 | 1 | 1 | ProQ[81..195] |
| YP_006781795.1 | 1 | 1 | HOK_GEF[6..47] |
| YP_006792580.1 | 1 | 1 | MazE_antitoxin[7..56] |
| YP_006792581.1 | 1 | 1 | PIN[9..135] |
